# 

**Published:** 1850-06

**Authors:** 


### INDEX TO VOLUME SIXTH.

Page.

Aisenic, Case of Poisoning by, and Analysis, by Prof. Hadley,..... 1
Anæsthetic agents, comparative value of,...... 22
Association, Am. Medical, annual meeting,...... 47
Association, American Medical, Prize offered by,...... 301
Association, American Medical, Dr. Warren's address,...... 381
Association, Am. Medical, Transactions of,...... 381, 401
Association of Superintendents of asylums for Insane, annual meeting of,...... 302
Association, Southern Central New York,...... 315
Ammonia, phosphate of, in Gout, and Rheumatism,...... 364
Amputations, statistics of, by Dr. Geo. Hayward,...... 368
Aphtha,...... 372
Assimilated Rank in Navy, 252, 373, 657
Apothecary, Boston, trial of,...... 384
" accident through carelessness of,...... 192
Anatomy, moral influence of study of, 246
Alcohol, use and abuse of, by Dr. Carpenter,...... 442
Abscesses, new way of opening,.... 479
Abscess, chronic, new mode of treating,...... 677
Asthma, nitric acid in,...... 680
Burns, treatment of,...... 103
Bell's Hydrology,...... 125
British American Med. Journal,.... 128
Blackwell, Miss, M. D.,...... 319
Buffalo Med. College,.... 320, 256, 273
" Graduates of,. 703
Bell, Dr. John, memorial,...... 375
Braithwaite's Retrospect,...... 381
Brinkerhoff, Dr. S. B., on application of collodion in small pox,...... 155
Bronchocele, treatment of, by compression,...... 468
Bryan, Prof. James, on Electro-Physiology,...... 513
Buffalo Hospital of Sisters of Charity, 574
Beck John B., obituary,...... 759
Buffalo Medical College,...... 761

Page.

Chorea, by Dr. Lee,...... 487
Cloasma,...... 488
Colon, secreting function of,...... 544
Chloroform on, by Prof. Gross,..... 559
Camp, Dr. John G., obituary,...... 702
Cold, as an anæsthetic agent,...... 750
Cupping with Tumblers to arrest vomiting,...... 733
Diarrhœa, chronic, case of, by C. Colegrove, M. D.,...... 13
Drake's Systematic Treatise,...... 62
Dysentery, injections of nitrate of silver in,...... 99
Dysentery, Epidemic, by Dr. Murphy,...... 619
Dysentery, treatment of, by Dr. Tullis,...... 673
Diathesis, hemorrhagic, cure of,.... 104
Dislocations, congenital, of femur,.. 116
Dissertations, prize, by Dr. Parsons, 123
Dwight, Dr. Wm. C., on treatment of Bronchocele by compression, 460
Dog, remedy for ferociousness of,... 48[illegible]
Deafness caused by tooth impacted in ear,...... 489
Dislocation of lower jaw, new mode of reducing,...... 489
Dunglison's Physiology,...... 509
Dunglison's Therapeutics,...... 512
Dislocation of humerus,...... 675
Diuretics, external use of,...... 679
Epilepsy, treatment of,...... 45
Eye, Semeiology of, by Prof. Lee,.. 65
Evolution spontaneous of fœtus,.... 106
Erie County Poor House,...... 377
Eberle on diseases of children,...... 382
Etherization, discovery of,...... 190
Editorial changes,...... 511
Electro-Physiology, by Prof. James Bryan,...... 513
Flint, Austin, M. D., contributions to study of Physical Diagnosis of Chest,...... 86
" Report on Continued Fever, 129, 193, 257, 321, 385, 449
" cases illustrative of effusion within arachnoid, as cause of sudden death,...... 404
" case of obstipation and stercoraceous vomiting,...... 530

Page.
Fractures, extension in, by adhesive plaster, ... 430
Fallopian pregnancy, case of, by Dr. Watkins, ... 592
Frick on renal affections, ... 701
Fevers, typhoid and typhus, identity of, by Dr. Jenner, ... 549
Fracture of leg to improve defective surgery, ... 561
Gall bladder, absence of, ... 297
Gazette Medical, of New York, ... 191
Griffith's Universal Formulary, ... 252
Gardner, Dr. Wm. H., case of paraplegia, ... 579
Hall, Marshall, and the spinal nervous system, ... 756
Hadley Prof., chemical examination of stomach in case of poisoning by arsenic, ... 1
Hamilton Prof., Report of unusual cases, ... 10
" Life of John Ellis Marshall, 282
" Surgical cases, ... 148
" on cataract and amaurosis produced by stramonium, ... 214
" Lithotomy on a female, ... 577
" Shoulder accidents, ... 641
" Translation of Amussat on the use of water in surgery, ... 705
Hydrocephalus Internus, case of, by C. Colegrove, M. D., ... 13
Hernia, anomalous case of, by Dr. Ring, ... 16
Hunt Dr. S. B., on absence of mammary development, ... 121
Homœopathia, anecdote, ... 299
Homœopathic Statistics, ... 313, 380
Homœopathy, Report upon by Mass. Med. Society, ... 685
" repudiated in law, ... 252
" correspondence of Dr. C. J. Williams, ... 255
Hydrometra, by Dr. Storer, ... 350
Head, passage of iron bar through, .. 165
Harris Prof. Chapin A., address, ... 249
Hullihen Dr. S. P., address, ... 249
Hubbard, Dr. Silas, theory of production of males and females, ... 252
" on Temperaments and constitutions, ... 584
Hæmorrhoids, Linseed oil in, ... 490
" internal, treatment of, .. 676
Hæmorrhage, uterine, new mode of arresting, ... 752
Hoyt Dr. Jonathan, decease of, ... 512
Insanity, hereditary, ... 46
" treatment of by warm and cold bathing, ... 539
Intermittents, common salt in, ... 752
Inoculation in rubeola, ... 731
Iron, ... 727
Iodine, injections of, in dropsical affections, ... 287
Page.
Jeffries Dr., Address, ... 191
Jarvis, on insanity in the sexes, ... 508
Kousso, ... 256
Knox, on races of men, ... 511
Labor premature, induced, by Dr. Blatchford, ... 30
Labor premature, case of, by Dr. Tyler, ... 169
Lee Prof. on the Semeiology of the eye, ... 65
" Biography of, ... 184
" on Epidemics, &c. of United States, ... 216
Lobelia inflata, two cases of poisoning with, ... 100
Louisville Medical College, ... 320
Lead, poisoning by, remarkable case of, ... 417
Lead, poisoning by, in New Orleans, 671
Liver, enlargment of, auscultatory sign, ... 478
Lord Dr. Asa, on Evidence and testimony, ... 667
Latin and Greek, importance of to the Physician, ... 737
Lithotomy, on a female, by Prof. Hamilton, ... 577
Midwifery, Demonstrative, ... 64, 175, 250, 378, 568
Midwifery, Demonstrative, trial for libel, ... 110
Midwifery Demonstrative, withdrawal of one of the ... 17, 380
Midwifery Demonstrative, and C. M., 448
" " ... 758
Medical Association of State of Alabama, proceedings of, ... 759
Mammary development, absence of, 121
Michigan Medical Association, ... 122
Marshall, Dr. John Ellis, Life of, ... 282
Mistakes, fatal, ... 299
Menstruation, vicarious, ... 302
Mitchell's Materia Medica, ... 382
Medical Journals, new, ... 383, 575
Medical Education, practical views on, by Medical Faculty of Harvard University, ... 172
Medical Jurisprudence, Dean's, ... 189
Midwifery, Cazeaux's, ... 190
Medicine, History of, by Prof. Bryan, 191
Medical Institutions, new, ... 192
Medical Education, report of Committee of American Medical Association, ... 408
Mange communicated by a pig, ... 431
McClintock's Prof., Introductory Lecture, ... 445
Medical Schools, Statistics of, ... 98
Matico, ... 476
Morceau, precious, ... 490
Meigs on diseases of children, ... 499
Maclise's surgical anatomy, ... 510
Medical appointments, ... 510

Page.
Mitchell Dr. John, on internal otitis, 654
Medicine, sense and genius in the study of.................... 682
Mott on ligature of iliac artery,..... 558
Manganese, therapeutical uses of,.. 738
Nævi Materni,.................... 107
Nævi, treatment by compression,.... 684
New Hampshire Journal of Medicine,........................ 248
New York Register of Medicine and Pharmacy,.................. 758
New York Medical Gazette,........ 761
New Jersey Reporter,............ 761
New York State Medical Society, semi-annual meeting of,....... 762
Ohio Medical and Surgical Journal,. 761
Ovarian Tumor, removed by Prof. March,...................... 30
Ovariotomy, case of,........... 40, 366
Ohio Medical College,............ 319
Os uteri, ulceration of,............ 355
" " rejoinder of Dr. Bennett,.. 360
" " rigidity of, treatment by tartar emetic,.................... 481
Obstetrical extractor, Dr. Evans,.... 161
" statistics,...........433, 466
" clinique,............... 511
Obesity, treatment of, by Dr. Chambers,......................... 614
Ophthalmia granular, treatment of by acetate of Lead,.............. 618
Otitis internal, extending to brain,.. 654
Ovaritis and puerperal mania, by Prof. Gilman, ................. 690
Obstipation, with stercoraceous vomiting, by Editor,............ 530
Operation, the mania for,.......... 735
Professorial appointments,......... 188
Pie plant, poisoning by,............ 256
Pulmonary gangrene, by Moreton Stille, M. D................. 413
Paraplegia, case of, by Dr. Gardner, 579
Paris correspondent, letter from,.... 644
Poisons, morbid, by Dr. Simon,.... 742
Pruritus of vulva in children,....... 751
Quinia, method of depriving of bitterness, ..................... 108
" mode of testing,........... 367
" history of, by Dr. T. S. Bell, 562
Quackery, 475,................... 475
Ring, Dr. Wm., report of case of hernia,...................... 16
Rodgers M. M., M. D., on application of chemistry to prac. Med.,. 80
" on modus operandi of medicines chemically considered,... 221
" on injection of stomach in gastritis,.................... 252
Rheumatism, quinine and opium in, 97
" treatment of, by Dr. Upshur, 353
" phosphate of ammonia in,.. 364
" treatment of, by lemon juice, 734
Ranking's Abstract,............... 381
Page.
Robinson Dr. James, address,....... 249
Rubeola, inunction in, by Prof. Evans 429
Revenge, singular,................ 429
Root Dr. John, on treatment of spermatorrhœa by cauterization,.... 470
Rochester knockings, discovery of the source of,............ 628, 698
Rochester Knockings,............. 762
Rogers S. C. Dr., Report of medical cases,...................... 646
Rowe Dr. Alfred Milton, obituary,.. 572
Respiration and air,............... 748
Strong, Dr. P. H., report of case of croup,...................... 17
Staten Island Quarantine Hospital Report, ..................... 124
Southern Medical Reports, by Dr. Fenner, ..................... 126
Schieffelin & Co's extracts,........ 128
Speculum, on use of, by Prof. Gilman 128
" vaginal,............... 378
Small Pox, application of collodion, 155
Shotwell Prof., decease of,......... 192
Sleep phrenologically considered by Dr. Fosgate,................. 249
Smith, I V. C., M. D.,............ 448
Spermatorrhœa treated by cauterization, ........................ 470
Sprains, treatment of,............. 486
Spectacles their use and abuses,.... 501
Sanitary Commission of Mass., Report of,...................... 596
Sanitary Institutions,.............. 753
Scarlatina, treatment by inunction,.. 610
" inunction in,............ 688
Shoulder accidents, by Prof. Hamilton 641
Small Pox, abortive treatment of, by application of collodion,........ 760
St. Louis Probe,.................. 760
Tannic acid, therapeutical effects of, 346
Taenia treated with Kousso,....... 369
Typhus or ship fever,............. 376
Taylor's Medical Jurisprudence,.... 383
Townsend, Dr. Elisha, address,.... 249
Treat Dr. Wm., case of anteversion during parturition,............ 401
Tannin,.......................... 476
Trachea, foreign body in,.......... 478
Temperaments and constitutions, by 478
Dr. Hubbard, ................ 584
Tuthill Dr., on registration of births, marriages, and deaths,........ 690
Twitchell, Amos, M. D., memoir of, 755
Urine, incontinence in, in children, 365
Uterus, extraction of, by Prof. Eve, 471
Urine, retention of, new mode of relieving, ...................... 689
Vaccine depot,................... 512
Venereal diseases, by Dr. Acton,.... 661
Veratrum Viride, in Fevers, etc.,... 741
Water, Dr. Gordon's Lectures on,.. 157
Williams, Dr. P. O., on obstetrical statistics, .................... 466

Watkins, Dr. W. H., case of Fallopian pregnancy,.............. 592
Water, use in Surgery, by Amussat, 705
Year, new,...................... 512

NOTE.—For original articles contributed for this Journal, look for names of contributors, as, also, the names of subjects. For other articles, except the bibliographical notices, look for names of the subjects. For notices of works, look generally for the names of the authors. When subjects relate to diseases, look generally for the name of the disease.

☞The notices of the meeting of the American Medical Association, and of the New York State Society, have excluded a notice of an address by Prof. Coventry, and several other articles prepared for the editorial department, which will appear in our next No.

